# A novel controller based on state-transition models for closed-loop vagus nerve stimulation: Application to heart rate regulation

**DOI:** 10.1371/journal.pone.0186068

**Published:** 2017-10-27

**Authors:** Hector M. Romero-Ugalde, Virginie Le Rolle, Jean-Luc Bonnet, Christine Henry, Alain Bel, Philippe Mabo, Guy Carrault, Alfredo I. Hernández

**Affiliations:** 1 INSERM, U1099, Rennes, F-35000, France; 2 Université de Rennes 1, LTSI, Rennes, F-35000, France; 3 Sorin CRM SAS (a LivaNova company), Clamart, France; 4 INSERM, UMR970 Paris Cardio-vascular Research Center, Paris, France, Assistance Publique-Hôpitaux de Paris, Department of Cardiology, Hôpital Européen Georges Pompidou, Paris, France, Paris Descartes University, PRES Paris Sorbonne, Paris, France; 5 CHU Rennes, Department of Cardiology and INSERM, CIC-IT 1414, Rennes, F-35000, France; University of California Los Angeles, UNITED STATES

## Abstract

Vagus nerve stimulation (VNS) is an established adjunctive therapy for pharmacologically refractory epilepsy and depression and is currently in active clinical research for other applications. In current clinical studies, VNS is delivered in an open-loop approach, where VNS parameters are defined during a manual titration phase. However, the physiological response to a given VNS configuration shows significant inter and intra-patient variability and may significantly evolve through time. VNS closed-loop approaches, allowing for the optimization of the therapy in an adaptive manner, may be necessary to improve efficacy while reducing side effects. This paper proposes a generic, closed-loop control VNS system that is able to optimize a number of VNS parameters in an adaptive fashion, in order to keep a control variable within a specified range. Although the proposed control method is completely generic, an example application using the cardiac beat to beat interval (RR) as control variable will be developed in this paper. The proposed controller is based on a state transition model (STM) that can be configured using a partially or a fully-connected architecture, different model orders and different state-transition algorithms. The controller is applied to the adaptive regulation of heart rate and evaluated on 6 sheep, for 13 different targets, using partially-connected STM with 10 states. Also, partially and fully-connected STM defined by 30 states were applied to 7 other sheep for the same 10 targets. Results illustrate the interest of the proposed fully-connected STM and the feasibility of integrating this control system into an implantable neuromodulator.

## Introduction

Vagus nerve stimulation (VNS) is an effective therapy for partial or pharmacologically refractory epilepsy [1, 2] and depression [3] and a potential therapeutic approach, under active clinical research in a number of other clinical applications, such as supraventricular arrhythmias and heart failure (HF) [4–6]. In heart failure therapy, two ways for delivering VNS exist. In one approach, VNS is delivered asynchronously, without any cardiac synchronization [7, 8]. In a second approach, VNS is timed to the cardiac cycle (synchronous VNS) [9, 10]. Although both approaches have already been clinically evaluated, they have failed to demonstrate clinical efficacy in HF patients [7–9]. This lack of clear clinical efficacy may be due to the fact that VNS parameter values are currently defined through a non-standardized manual titration phase. The interest of an automatic, closed-loop method for VNS parameter configuration is clear in this context.

Closed-loop VNS methods have been proposed for the asynchronous case, and have been experimentally evaluated [11–15]. However, these methods show a number of limitations. Firstly, as mentioned above, none of these works handle synchronous VNS. In fact, the definition of a closed-loop control for cardiac-synchronized VNS requires an adaptive discrete-time controller, able to deal with irregular sampling rates, since the cardiac period is not constant. Novel controllers have to be developed in this sense. Secondly, these controllers are restricted to the modulation of a single VNS parameter (amplitude or frequency of the delivered current), even though recent experimental studies have shown that a variety of parameters should be jointly modulated in order to correctly optimize VNS [16, 17]. Finally, the complexity of some of the previously proposed methods makes difficult a real-time integration into an implantable device.

To our knowledge, only our group has worked in overcoming these obstacles, by developing closed-loop synchronous VNS methods, evaluated in experimental conditions [18–20]. In a first approach [20], we presented an “on-off” controller, that provided a simple and robust control, but led to strong heart rate oscillations. In order to reduce these oscillations, we implemented and evaluated a proportional-integral (PI) controller [18, 19]. Control performance was significantly improved, but the obtained performance was highly dependent on the PI control parameters and optimal, subject-specific values for these parameters were difficult to define. A model-based design approach was also proposed by our group, to find the best PI parameter ranges and simplify subject-specific parameter definition [18]. However, as in other previous works, the obtained PI controller was limited to the modulation of only one VNS parameter (amplitude of the delivered current).

In this paper, we propose and characterize a novel VNS controller, based on a state transition model (STM), which is able to modulate multiple VNS parameters as a function of the patient state and has a level of complexity suitable to be embedded into an implantable medical device. In order to characterize the proposed controller, experimental evaluations on sheep are applied using different controller configurations. In particular, we evaluate the influence on control performance of the STM architecture (partially and fully-connected), the model order and the state-transition method.

The paper is organized as follows: Section 1 presents a detailed description of the proposed closed-loop control system and the evaluation methodology, which includes an experimental protocol. In section 2, results of the proposed STM-based controller are presented and the performance associated with the different configurations are compared. Discussion on the main findings and limitations of the study are given in Section 3.

## 1 Methods

Data for this study were obtained from healthy sheep following a protocol approved by the French local ethics committee for animal experimentation (“Comité d’éthique en matière d’expérimentation animale”, Paris Descartes, Paris, France).

### 1.1 General description of the proposed closed-loop control system

**Fig 1:**
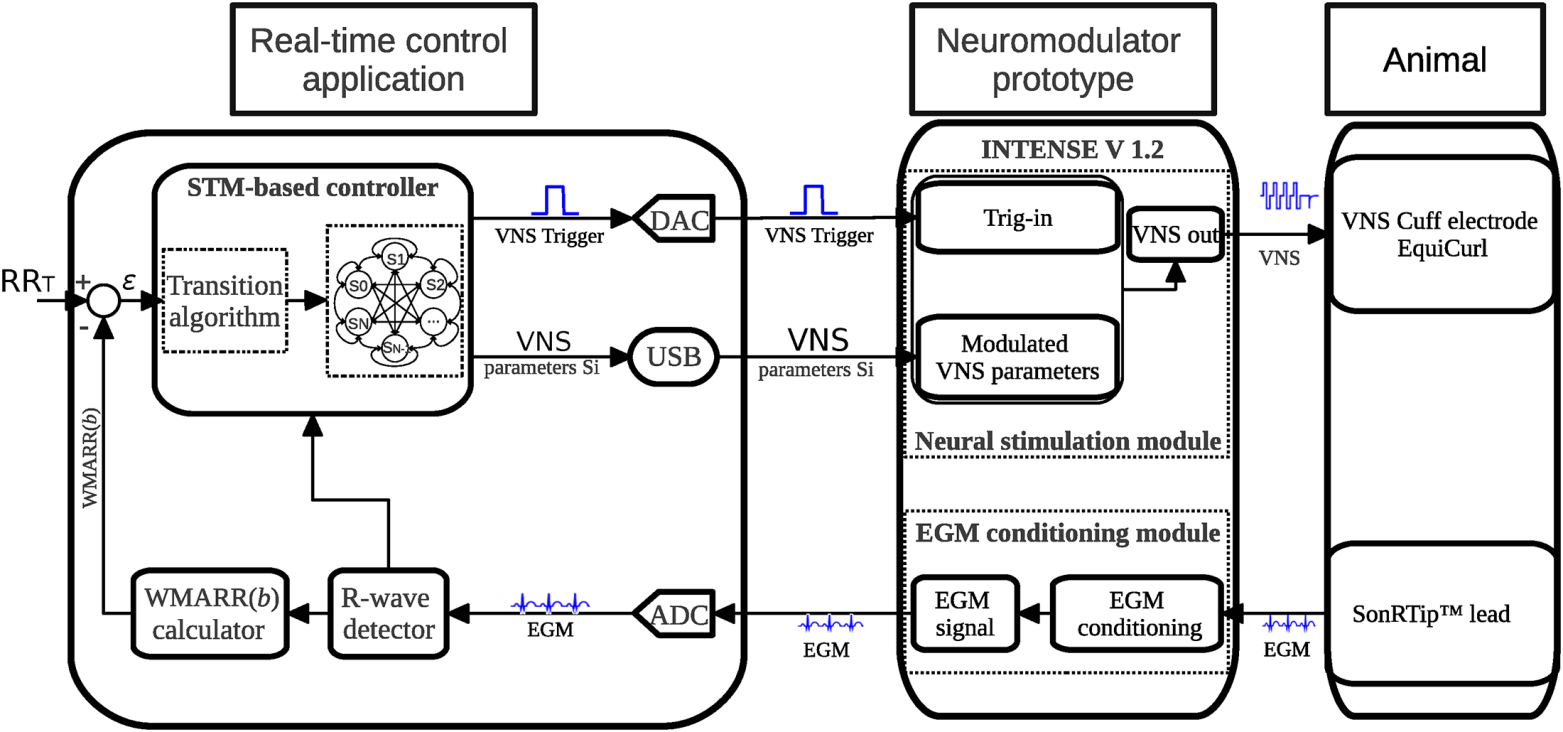
Proposed closed-loop control system, based on a fully-connected state transition model (STM). The system consists of three components: a real-time control application, a neuromodulator prototype and a sheep used for animal experimentation. An intracardiac electrogram (EGM) is obtained via a cardiac lead implanted into the right ventricle of the sheep, analogically processed by the neuromodulator, and acquired via the analog to digital converter into the real-time control application. R-wave instants are detected and used for calculating a Weighted Moving Average of the RR intervals WMARR(b), which will be used as control variable. An error *ϵ* is obtained from the difference between the target (RR_T_) and WMARR(b). The STM-based controller estimates a new set of VNS parameters *S*_*i*_, minimizing *ϵ*. VNS is triggered synchronously to the R-wave, with the new set of parameters *S*_*i*_, to deliver VNS to the right vagus nerve of the sheep. VNS with these new parameters will modify the acquired EGM and a weighted moving average of the new RR interval is computed, closing the loop.

The objective of the proposed control system is to adaptively modulate the VNS parameters of a neuromodulator in order to maintain a physiological control variable, observed in real time from a patient, within a given target range. In this paper, the cardiac beat to beat (RR) interval will be used as control variable for evaluation purposes, since current studies on open loop VNS therapies use the heart rate as the main reference for titration [7–9]. Fig 1 shows the proposed closed-loop control system, combining i) a custom-made real-time control application, ii) a neuromodulator prototype (INTENSE v1.2) and iii) a sheep, for animal experimentation.

The control application is composed of an analog to digital converter (ADC), a digital to analog converter (DAC), a cardiac R-wave detector module, a control variable calculator (WMARR(b) calculator) and an STM-based controller. An intracardiac signal observed from the animal by the SonRTip^™^ lead (Sorin Group Italia, Saluggia, Italy), analogically preprocessed by the EGM conditioning module of the neuromodulator prototype, is acquired via the ADC and processed by an R-wave detector, which provides R-wave time instants for each detected beat *b*. The control variable calculator module computes a Weighted Moving Average of the RR interval, WMARR(b). Based on WMARR(b) and a target RR (RR_T_) given by the user, an error *ϵ* is calculated and presented as input to the proposed STM controller. This controller seeks to find the set of VNS stimulation parameters *S*_*i*_ minimizing *ϵ*. The new VNS parameters *S*_*i*_ are programmed into the neuromodulator via USB communication. After parameter modulation, the controller determines the instant of VNS activation (P_del_), using the currently detected R-wave instant as a reference. The VNS activation instant is transmitted, via a squared pulse analog signal (VNS Trigger), to the Trig-in input of the neuromodulator by means of the DAC. The new WMARR(b) is computed. More details on the WMARR(b) computation and the STM-based controller are given below.

The neuromodulator (Prototype INTENSE v1.2, Sorin CRM, Clamart, France) is composed of an EGM conditioning module and a neural stimulation module with a dynamic programmable interface, allowing for the generation of complex temporal stimulation waveforms [21]. Details on animal experimentation and how each sheep is instrumented are given in section 1.5.

### 1.2 VNS parameters

**Fig 2:**
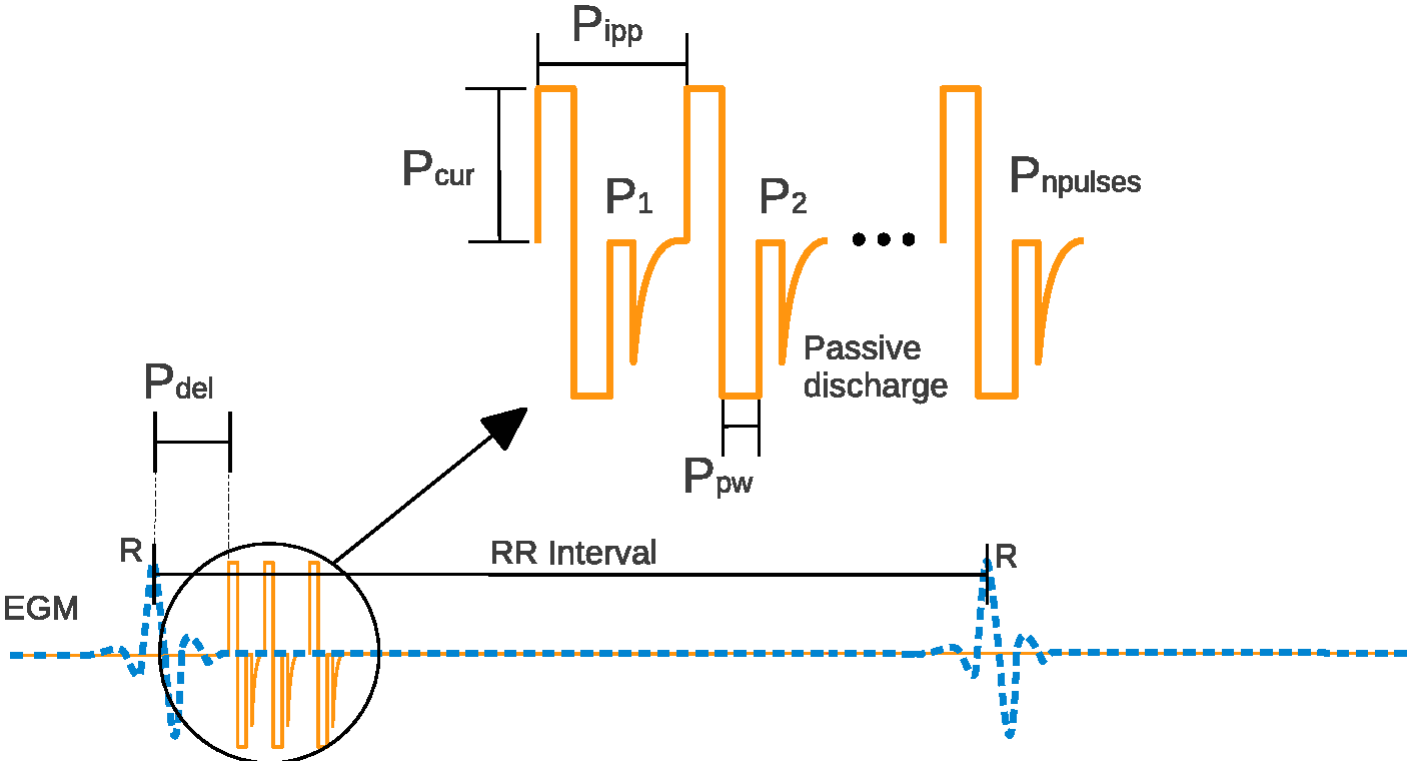
Typical vagus nerve stimulation pattern (solid line), synchronized with the cardiac activity (dashed line). The controller can modulate the following VNS parameters: number of pulses (*P*_npulses_), the interpulse period (*P*_ipp_, ms), delay (*P*_del_, ms), current amplitude (*P*_cur_, mA), and pulse width (*P*_pw_, ms).

A cardiac-synchronized vagus nerve stimulation burst is composed of biphasic pulses, delivered after a programmable preset delay from the last detected R-wave, and characterized by a set of VNS parameters (Fig 2). The VNS parameters modulated by the proposed STM-based control algorithm are: the number of pulses in the train of stimulation (*P*_npulses_), the interpulse period (*P*_ipp_, ms), the delay between cardiac event and VNS onset (*P*_del_, ms), the current amplitude (*P*_cur_, mA), and the pulse width (*P*_pw_, ms).

The parameter ranges, predefined according to our previous experience on sensitivity analysis of the chronotropic effect with respect to the above-mentioned VNS parameters [16, 17], are: *P*_npulses_ ∈ [1, 4], *P*_ipp_ ∈ [23.4, 46.9] ms, *P*_del_ ∈ [16, 156] ms, *P*_cur_ ∈ [0.2, 1.0] mA, and *P*_pw_ ∈ [0.05, 0.20] ms. These parameter ranges will be used throughout this manuscript for VNS control.

### 1.3 Computation of the control variable

The instantaneous RR interval (RR(b)) is constantly modulated by the autonomic nervous system and other physiological factors. Indeed, arrhythmic events may occur, inducing a wrong response of the controller. To reduce consequences of these arrhythmic events on the controller, we propose to use WMARR(b) as control variable instead of the instantaneous RR(b). WMARR(b) is a weighted moving average, computed online over the last four RR intervals, according to Eq (1).

$$\begin{array}{c} \text{WMARR(b)}=\frac{\sum_{i=0}^{3}(\beta_{i}\text{RR}\left(\text{b}-i\right)}{\sum_{i=0}^{3}\beta_{i}} \end{array}$$ (1)

The weights *β*_0_ = 0.98 > *β*_1_ = 0.40 > *β*_2_ = 0.10 > *β*_3_ = 0.01 were heuristically chosen as a good compromise between smoothness and reactivity.

### 1.4 State transition model-based controller

An STM consists of a set of states and behaviors [22]. The STM-based controller proposed in this paper is composed of a state-transition model associated with a state transition algorithm (STA). Only a single state can be active at a given time (current state). A transition from one state to another is performed by a triggering event or condition (transition algorithm) in order to perform different actions. A more detailed description of state transition models can be found elsewhere [23].

Two different STM-based controllers are evaluated in this study. The first one is based on a partially-connected STM, with its corresponding partially-connected STA. The second controller is based on a fully-connected STM with its corresponding fully-connected STA. The partially-connected STA allows for transitions only between neighbor states of the partially-connected STM, whereas the fully-connected STA allows for transitions between all the states of the STM.

#### 1.4.1 State transition model

**Fig 3:**
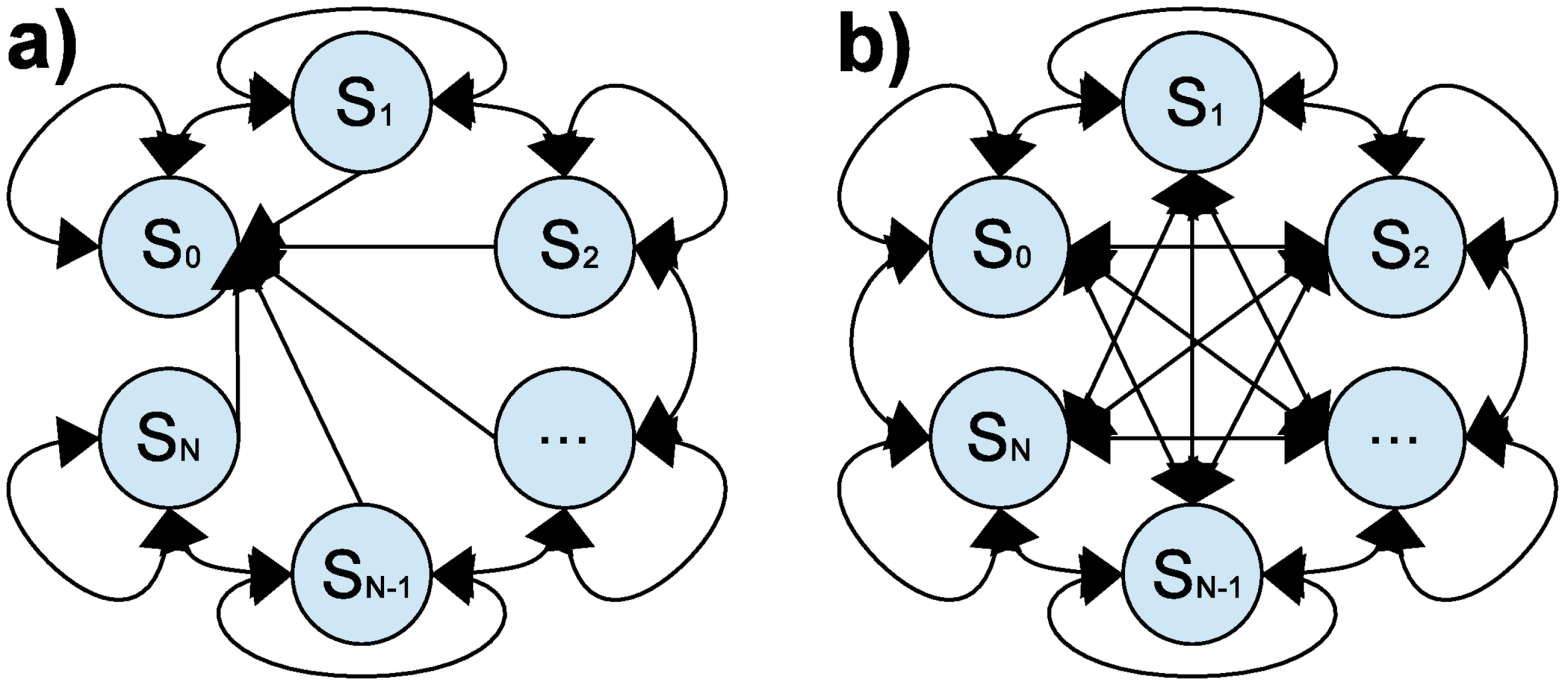
a) Partially-connected state transition model of *N* + 1 states. The current state (*CS*(*b*) = *S*_*i*_) may transit to a neighbor state (*S*_i-1_ or *S*_i+1_) or stay at the same state (*S*_i_). All states are connected to state *S*_0_, which turns off VNS. b) Fully-connected state-transition model of *N* + 1 states. The current state (*CS*(*b*) = *S*_i_) may transit to any state (*S*_i-k_ or *S*_i+k_) or stay at the same state (*S*_i_). All states are connected with all the states including the state *S*_0_, which turns off VNS.

Fig 3 shows the partially- (Fig 3(a)) and the fully- (Fig 3(b)) connected STMs on which the proposed controllers are based. In both STM-based controllers, the STM is composed of *N* + 1 states (*S*_0_,…,*S*_N_) and each state is associated with a set of VNS parameters:

$$\begin{array}{c} S_{i}\;=\;\left[P_{\text{cur}},P_{\text{npulses}},P_{\text{pw}},P_{\text{ipp}},P_{\text{del}}\right], \end{array}$$ (2)

The states are ranked as a function of the effect they produce on the control variable when applying the corresponding VNS parameters during a training phase (see section 1.4.3). In this sense, the effect of state *i* − 1 (when applying VNS parameters *S*_*i*−1_) on the control variable is lower than the effect of state *i* (when applying VNS parameters *S*_*i*_), for *i* = 0, …, *N*. Both STMs include a non-stimulation state (*S*_0_) allowing the controller to turn off (no-stimulation) the VNS at any moment.

The difference between the partially- and fully-connected STMs consists in the possible transitions allowed between states. In fully-connected STMs, the current state (CS(b) = *S*_*i*_) is allowed to transition to any state:

$$\begin{array}{c} CS\left(b+1\right)\in \left\{S_{i}-k,\;S_{i}+k,\;S_{i},\;S_{0}\right\} \end{array}$$ (3)

where *k* is obtained as a function of the error between the target RR and the observed WMARR(*b*). In this paper, *k* is defined as the integer division of the error by the constant *δ* defined by the user:

$$\begin{array}{c} k=|\epsilon |/\delta \text{,}\;\text{with}\; \end{array}$$ (4)

$$\begin{array}{c} \epsilon =\text{RR}{}_{\text{T}}-\text{WMARR(}\text{b}\text{)}. \end{array}$$ (5)

Partially-connected STMs correspond to the particular case where *k* = 1, i.e., the states are only allowed to transition to a neighbor state.

#### 1.4.2 State transition algorithm

**Fig 4:**
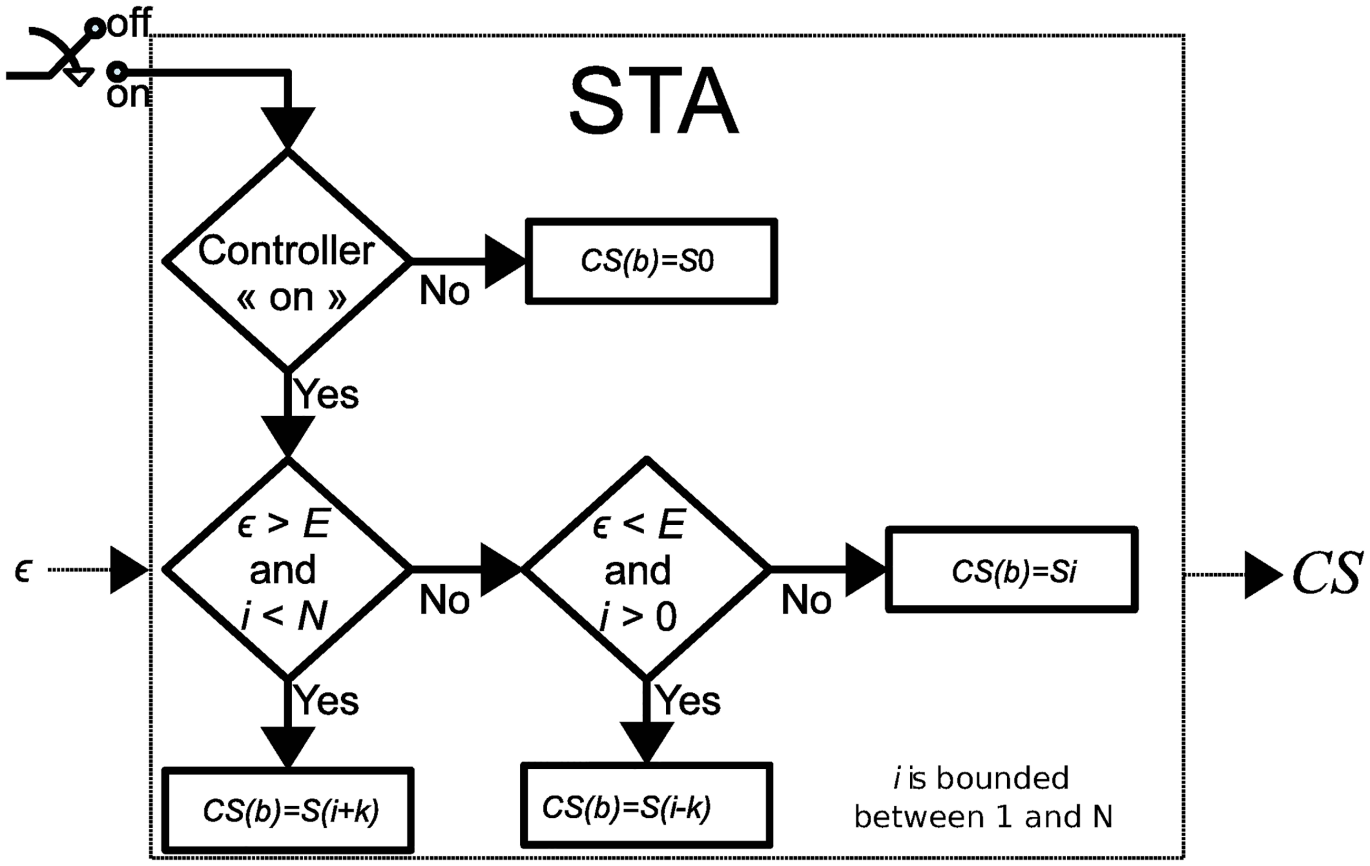
State-transition algorithm. The STA determines the optimal VNS parameters *S*_*i*_ minimizing the value *ϵ*, which represents the difference between the target RR and WMARR(b). Variable *i* is bounded between 0 and *N*. Parameter *E* defines the acceptable level of error on *ϵ*.

At each event *b*, the STA determines the state transition that minimizes the error between RR_T_ and the control variable WMARR(b). Fig 4 summarizes the algorithm that governs the STA behavior. When the controller is operating (ON), the next state is determined as a function of user-defined thresholds *E* (see Fig 4) that specifies the minimal amount of acceptable error *ϵ* between the target RR and the control variable.

The difference between the fully- and partially-connected STAs is mainly related to the definition of parameter *k*, which is essential to improve the time of convergence. In fact, the partially-connected STM-based controller will take *N*events to reach the state *S*_N_ whereas the fully-connected will take around (*N*/*k*)<N events to reach *S*_N_. Increasing the number of states *N* should lead to an improvement of the accuracy because differences between the responses associated with consecutive states are reduced.

#### 1.4.3 Training phase

During the training phase, the STM is firstly initialized with a set of population-based VNS parameters, which were obtained from our previous studies on stimulation parameters [16, 17]. The subject-specific training phase consists of:

1. stimulating the vagus nerve of the subject intra-operatively, by using each set of VNS parameters of the initialized STM;
2. measuring the corresponding effect (ΔRR_*i*_), evoked by VNS parameters on each state *S*_*i*_:
  $$\begin{array}{c} \Delta {\text{RR}}_{i}=\frac{{\bar{RR}}_{baseline}\left(S_{i}\right)-{\bar{RR}}_{stim}\left(S_{i}\right)}{{\bar{RR}}_{baseline}\left(S_{i}\right)} \end{array}$$ (6)
  where ${\bar{RR}}_{stim}\left(S_{i}\right)$ is the mean RR interval during VNS with parameters *S*_*i*_, and ${\bar{RR}}_{baseline}\left(S_{i}\right)$ is the mean RR interval, measured during a period of non-stimulation;
3. sorting states of the STM in ascending order, according to ΔRR_*i*_, in the range [ΔRR_min_, ΔRR_max_], where ΔRR_max_ is the effect provoked by the state *S*_*N*_ and ΔRR_min_ is the effect provoked by the state *S*_1_.

### 1.5 Experimental evaluation

#### 1.5.1 Experimental protocol for sheep preparation

The experimental study was conducted under the approval issued by the French ethics committee for animal experimentation. Sheep were initially anesthetized (induction) by propofol (4mg kg-1 min-1). Surgery for material implantation was performed under inhalation of isoflurane (1.5%), and control evaluation tests were performed under etomidate (100μg kg-1 min-1). A pressure and electrical probe sensor (Millar Instruments Inc., Houston, USA) was implanted into the left ventricle for pressure monitoring. A bipolar pacemaker lead containing an intracardiac accelerometer (SonRTip^™^ lead) was placed into the right ventricle, in order to acquire intracardiac electrograms (EGM). A quasitripolar cuff-type VNS electrode (Cuff electrode EquiCurl, Sorin Group Italia, Saluggia, Italy) was implanted on the right vagus nerve, at a cervical site. The carotid artery dissection is realized at an equal distance between the head and the trunk. The vagus nerve (VN) is gently removed from the carotid sheath and the cuff is placed around the VN. These leads were connected to the prototype neuromodulator, as shown in Fig 1.

After a verification stage of the implanted instrumentation (in particular VNS electrode impedance and EGM quality), the experimental evaluation of the proposed control system was performed. The surface ECG, the EGM, the left intra-ventricular pressure, and the body temperature were monitored during the whole procedure. Breathing was artificially controlled at 0.3 Hz (18 breaths min-1)using a Datex-Ohmeda Aestiva/5 anesthesia ventilator (GE Healthcare, Madison, Wis). This controlled ventilation also allowed us to maintain a stable respiratory sinus arrhythmia, which can be considered in this context as noise for the closed-loop control system.

#### 1.5.2 Control evaluation tests

The proposed experimental evaluation tests aim to quantify the performance of the partially-connected and fully-connected STM-based controllers, and to evaluate the effect obtained by i) increasing the number of states in the partially-connected STM and ii) introducing the fully-connected state-transition algorithm. Three different controllers were implemented and compared:

1. P_10_: partially-connected STM with *N* = 10 (11 states),
2. P_30_: partially-connected STM with *N* = 30 (31 states) and
3. F_30_: fully-connected STM with *N* = 30 (31 states).

Each controller is tested in a classical control test, consisting in turning on the controller during 1 min, after 1 min of resting period, without stimulation. Performance indicators described in section 1.5.3 are computed for each control test.

In order to measure the effect of increasing the number of states in partially-connected STM, P_10_ and P_30_ controllers were compared. P_10_ was used to regulate heart rate on 6 sheep for 13 different targets and P_30_ was applied to 7 sheep, for 10 different targets. For 3 sheep of the 7 included in the present work, both the P_10_ and P_30_ controllers could be tested.

To show the improvements achieved by implementing the fully-connected STA, a P_30_ controller and an F_30_ controller were used to regulate heart rate on the same 7 sheep for the same 10 targets. The *N* = 30 controllers P_30_ and F_30_ were exactly the same for a given sheep. The fact of using the same number of states in both controllers to reach the same targets on a given sheep, allowed us to analyze improvements achieved by this new fully-connected STA.

#### 1.5.3 Performance indicators

In order to measure the performance of the controller, the following indicators are computed: i) the mean squared error (MSE) during steady-state, ii) the percent overshoot (%OS), expressed as a percentage of the steady-state value, and iii) the number of beats required for the waveform to go from 10% of the final value to 90% of the final value, namely B_*r*_.

B_*r*_ is equivalent to the classical performance indicator “time rise”, but is given in number of beats instead of time (in seconds). In this way, comparisons (in terms of speed of convergence) between controllers are not dependent of the instantaneous RR interval. Remember, that the proposed STM-based controller uses beat detections instants to activate a transition (synchronous VNS approach).

MSE is calculated from the beat in which the system arrived to the steady state (*b*_*s*_), and over a temporal support of *M* beats, as follows:

$$\begin{array}{c} \text{MSE}=\frac{1}{M}\sum_{b=b_{s}}^{b_{s}+M}\epsilon^{2}\left(b\right) \end{array}$$ (7)

In this paper, *M* was set equal to 1 min in order to evaluate accuracy on a sufficiently long temporal support. In this sense, the proposed MSE is an quantitative indicator of the stability of the response, being higher for responses showing higher oscillations with respect to the expected target. Moreover, the “key performance indicator” KPI proposed in Romero et al. [18], which is a linear combination of the 3 above-mentioned indicators, is computed as follows:

$$\text{KPI}=0.25\frac{\bar{\text{MSE}}}{\text{max}\left(\bar{\text{MSE}}\right)}+0.25\frac{\bar{\%\text{OS}}}{\text{max}\left(\bar{\%\text{OS}}\right)}+0.5\frac{\bar{{\text{B}}_{r}}}{\text{max}\left(\bar{{\text{B}}_{r}}\right)}$$ (8)

where $\bar{\text{MSE}}$, $\bar{\text{\%OS}}$, and $\bar{{\text{B}}_{r}}$ are the mean values of the performance indicators evaluated on all sheep. Note that KPI provides a global evaluation criterion giving the same importance to the accuracy (MSE, %OS) and speed of convergence B_*r*_.

For analysis purposes, unpaired (P_10_ vs P_30_ and P_10_ vs F_30_) and paired (P_30_ vs F_30_) Wilcoxon tests were performed to measure statistical differences between the three controllers on the four performance measures. p-values between the three controllers are computed on the 4 proposed performance measures.

## 2 Results

**Fig 5:**
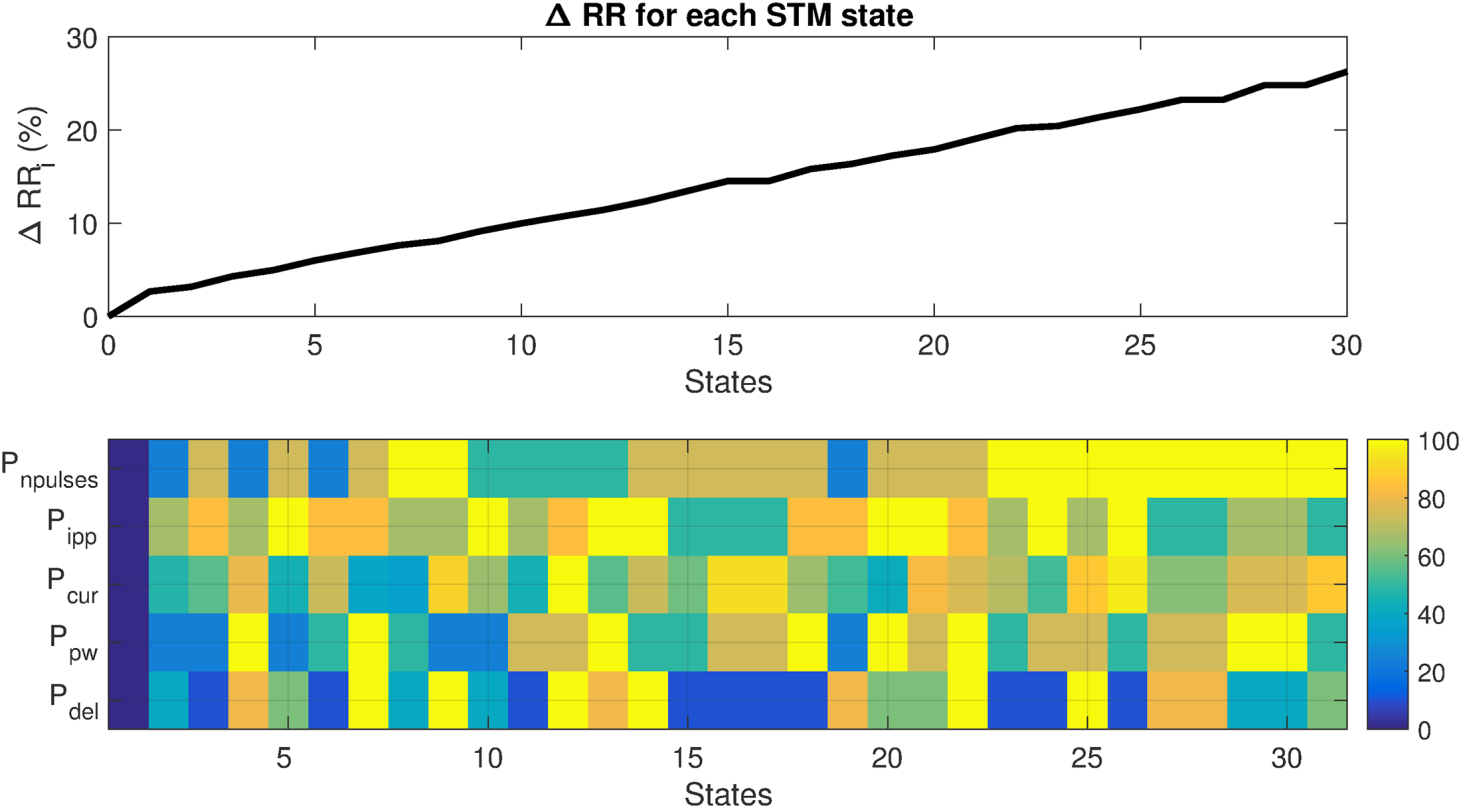
Example output of the training phase for a particular sheep and *N* = 30. Top panel: ΔRR_i_ as a function of sorted states. Bottom panel: Colormap of the normalized VNS parameters associated with each state, expressed as percentage of their corresponding range values.

Fig 5 shows a representative example output of the training phase for one particular subject. The upper panel of Fig 5 shows the observed ΔRR_i_ as a function of the STM states, once these states are sorted with respect to their response. The lower panel of Fig 5 displays a graphical representation of the normalized VNS parameters corresponding to each state. By definition, all parameters are set to 0 for *S*_0_ (VNS is off) leading to a ΔRR_i = 0_. Note that different combinations of the 5 VNS parameters are found for STM states. A similar approach has been used to define states in the case of *N* = 10. The output of the training phase was different for every sheep, both in the response of ΔRR_i_ and in the distribution of VNS parameters. This is in accordance to our findings published in [17], showing the usefulness of the definition of subject specific VNS parameters.

**Fig 6:**
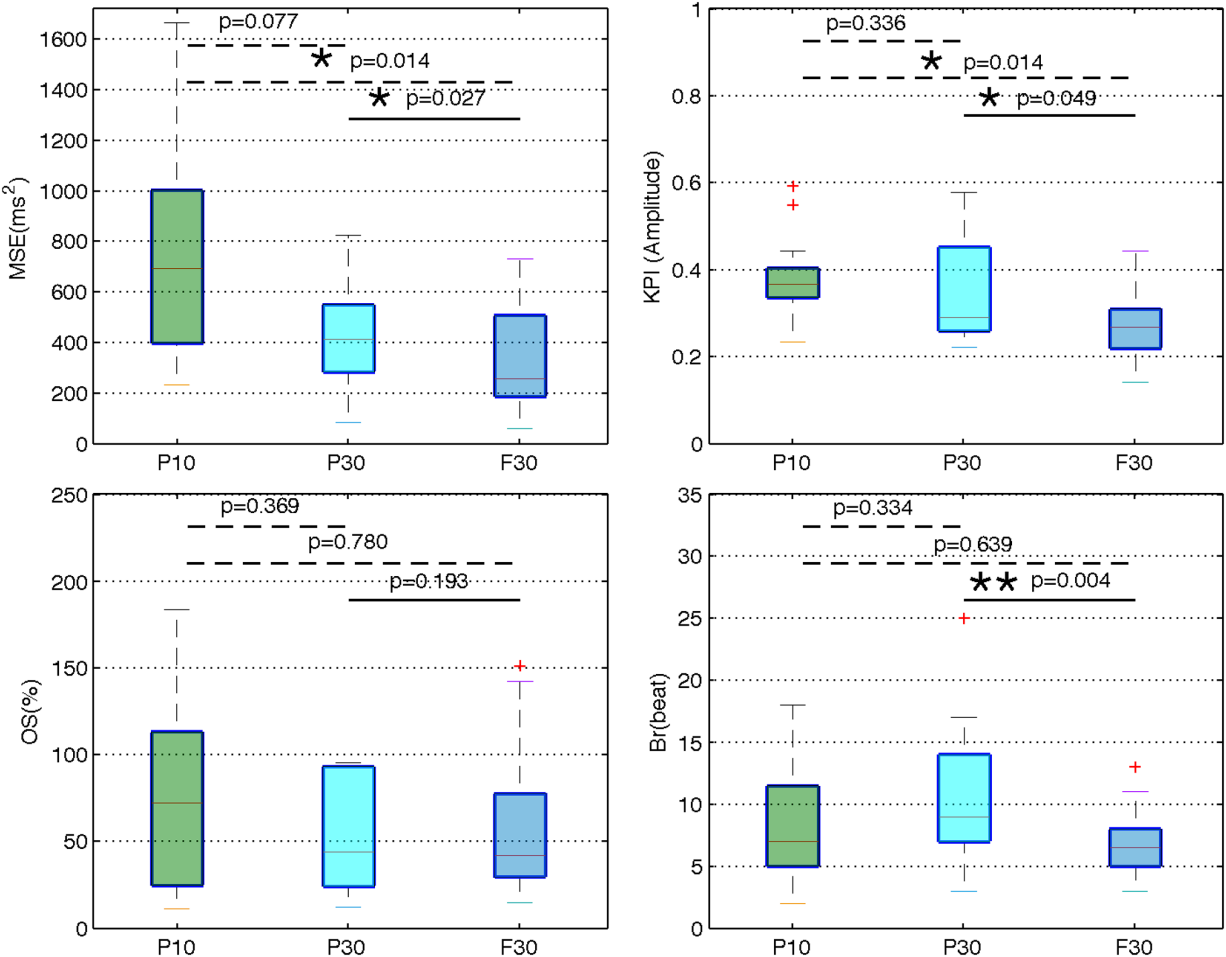
Performance indicators of three STM-based controllers. P_10_ is a partially-connected STM-based controller of *N* = 10 states, evaluated on 6 sheep for 13 different targets. P_30_ is a partially- connected STM-based controller of *N* = 30 states, evaluated on 7 sheep for 10 different targets. F_30_ is a fully-connected STM-based controller of *N* = 30 states evaluated on the same 7 sheep for the same 10 targets. The three controllers were used on sheep anesthetized by etomidate. Most of the sheep used in the experimental protocol P_10_ are different from the sheep used in the control tests of the P_30_ and F_30_ controllers. The sheep involved in the P_30_ and F_30_ protocols were the same. p-values are calculated between the three controllers. In the box plots, the dotted lines represent a Wilcoxon unpaired test and the solid line represents a Wilcoxon paired test. * denotes P < 0.05, and ** denotes P < 0.01.

After the application of the training phase, experimental evaluation of different configurations of the proposed STM controller have been performed and the performance indicators have been calculated in each case. Fig 6 shows box plots of these performance indicators, obtained for all sheep for the 3 controller configurations. This figure shows i) the effect of the number of states in the partially-connected STM, in terms of control performance and ii) the difference, in performance, between the fully- and partially-connected STAs. The dashed line represents a Wilcoxon unpaired test, performed between the two controllers that are linked by it, whereas the solid line represents a Wilcoxon paired test. p-values less than 0.05 are marked by *. p-values less than 0.01 are marked by **. In terms of accuracy, the median MSE obtained from the P_10_ controller is 693 ms^2^, for the P_30_ controller is about 410 ms^2^, and for the F_30_ controller is about 250 ms^2^. Concerning the %OS, the P_10_ controller obtains a median of 72%, the P_30_ controller obtains 44% and the F_30_ controller obtains about 42%. In terms of speed of convergence, the P_10_, P_30_ and F_30_ controllers obtain respectively median B_*r*_ values of 7, 9 and 6 beats. Finally, in terms of KPI, which is an indicator giving the same importance to the accuracy and the speed of convergence, the P_10_, P_30_ and F_30_ controllers obtain respectively median values of 0.36, 0.29, and 0.26.

Table 1 summarizes results on the comparison of the P_10_ and P_30_ controllers evaluated on 3 common sheep for the same targets. The mean MSE of the P_10_ controller is 665 ms^2^ whereas the P_30_ controller obtains a mean MSE of 227 ms^2^. The mean %OS of the P_10_ controller is 95% whereas the P_30_ controller obtains 82%. In terms of B_*r*_ the P_10_ controller obtains 8 beats whereas the P_30_ controller obtains 12. Finally, in terms of KPI, the mean value obtained by the P_10_ controller is 0.49 and the mean value obtained by the P_30_ controller is 0.43.

**Table 1:** P_10_ vs P_30_.

Sheep	Target(ms)	Controller	MSE(ms^2^)	%OS(%)	B_*r*_(beats)	KPI
1	460	P_10_	856	71.9	7	0.49
		P_30_	395	42.8	8	0.34
	600	P_10_	756	20.8	6	0.32
		P_30_	280	12.2	10	0.31
2	430	P_10_	815	183.4	11	0.71
		P_30_	180	197.9	23	0.80
3	430	P_10_	274	104.3	8	0.37
		P_30_	51	75.3	7	0.26

**Fig 7:**
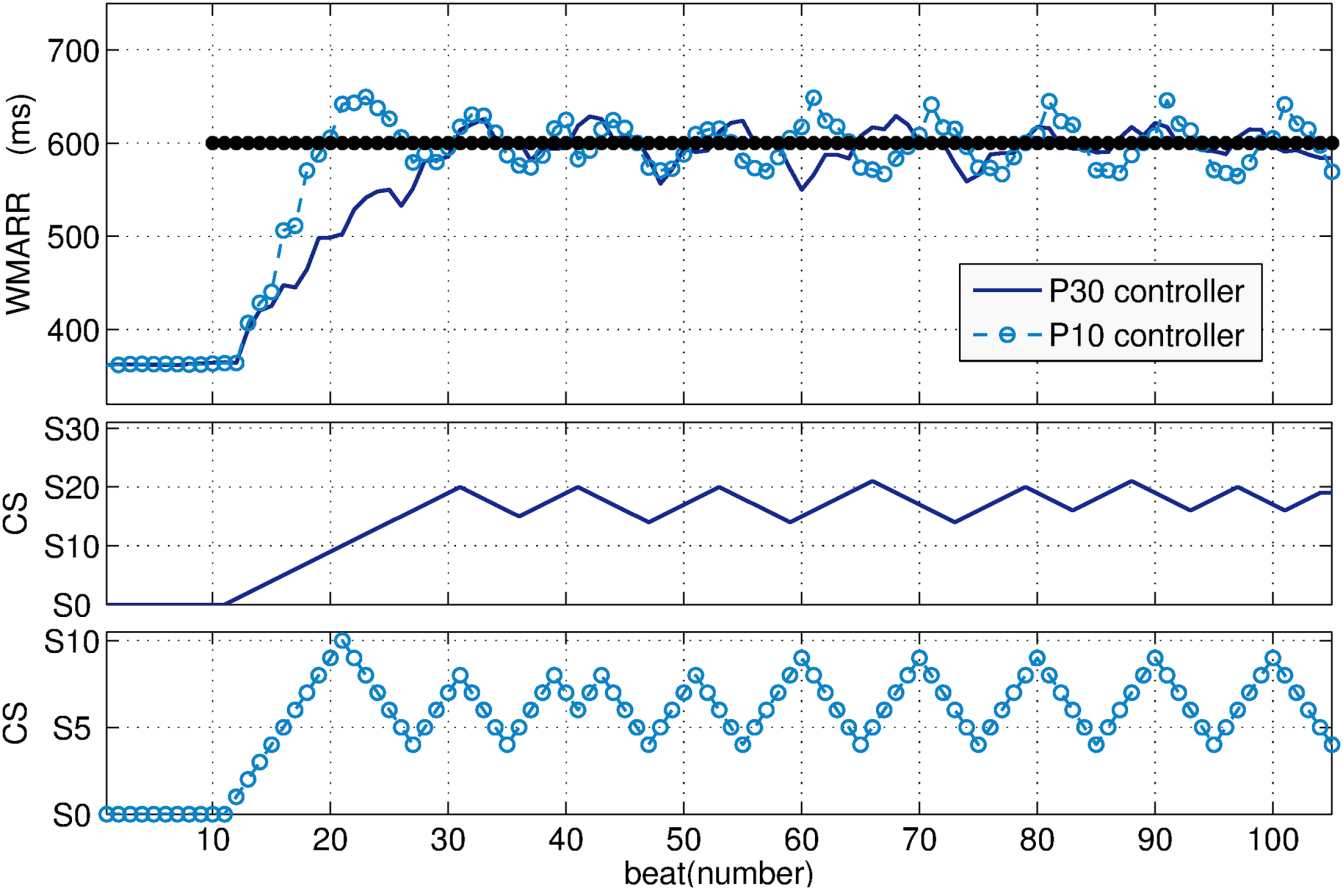
The top of the figure shows the WMARR response when P_10_ (dotted line) and P_30_ (solid line) controllers are used to regulate RR to 600 ms. The middle shows the dynamics of the P_30_. The bottom shows the dynamics of the P_10_ controller.

Fig 7 displays an example of the comparisons between the P_10_ and P_30_ controllers (Table 1, Sheep 1). The top panel of this figure shows the control variable WMARR(b) when the P_10_ (dotted line) and the P_30_ (solid line) controllers are used to regulate cardiac cycle length at a target RR set to 600 ms. With both controllers the target was reached. As expected, the P_10_ controller leads to a faster response (reduced B_*r*_), however, %OS and MSE are increased.

The middle and bottom panels of Fig 7, respectively, show the state transitions of the P_10_ and P_30_ controllers, to reach such target. The P_30_ controller oscillates among states S_14_ and S_21_ (see Fig 7 (middle)), whereas the P_10_ controller oscillates among states S_4_ and S_9_ (see Fig 7 (bottom)). This implies that, the P_30_ controller requires 14 transitions to reach the minimum state leading the steady state, whereas the P_10_ controller only requires 4 transitions.

**Fig 8:**
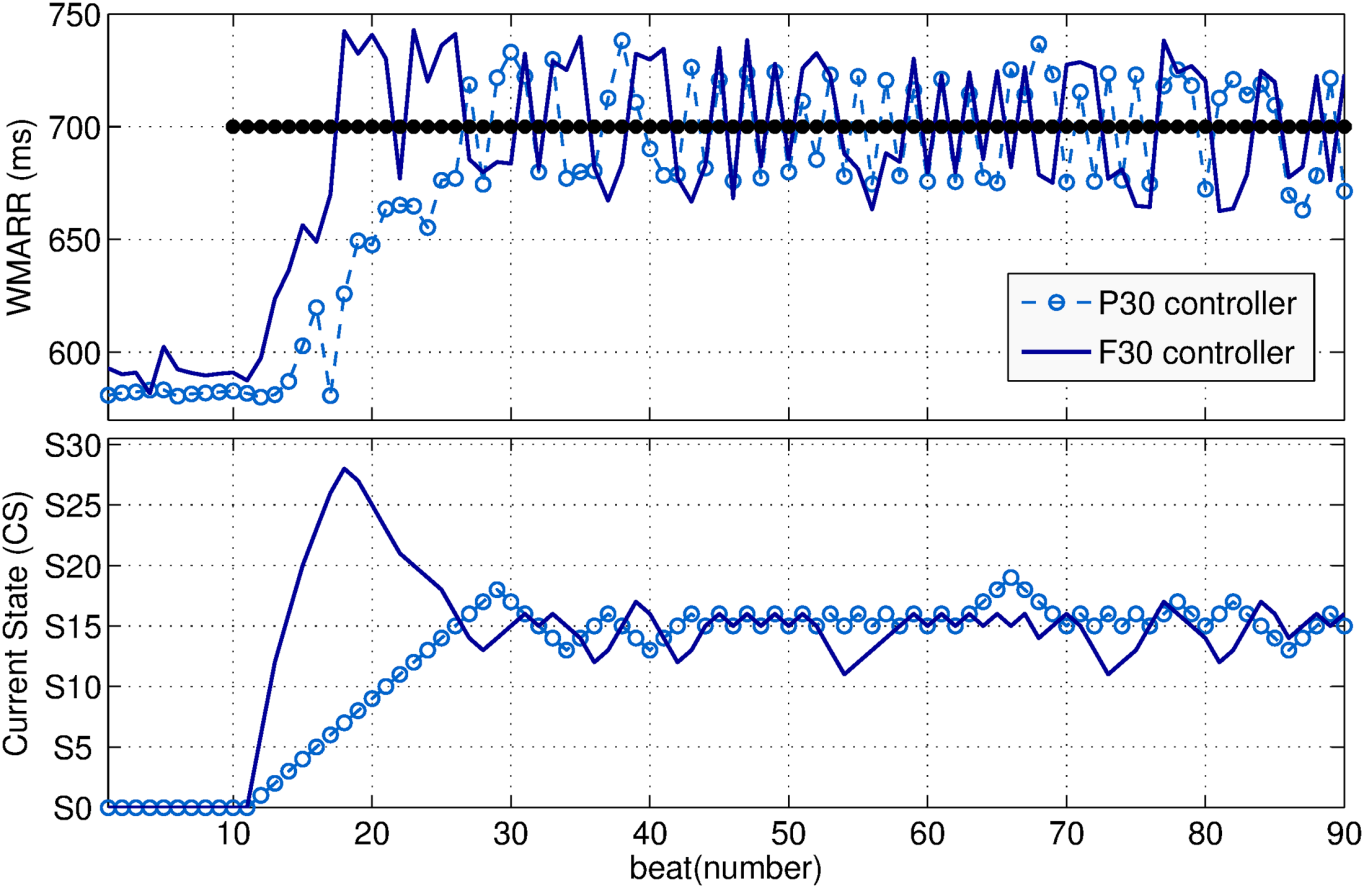
The top panel of the figure shows the WMARR response when the P_30_ (dotted line) and F_30_ (solid line) controllers are used to regulate RR to 700 ms. The bottom panel shows the dynamics of the P_30_ (dotted line) and F_30_ (solid line) controllers.

Fig 8 shows an example of the comparison between the P_30_ (dotted line) and F_30_ (solid line) controllers, for heart rate regulation on the same sheep for the target RR set to 700 ms. The top panel of this figure shows the response of the control variable WMARR. The bottom panel shows the state transitions of both controllers to reach such target. Since in both controllers the STM are composed of exactly the same *N* = 30 states, the current state CS tends to state S_15_ which may be the optimal state in this case. The F_30_ controller reached the target faster than the P_30_ controller. This was expected, since the fully-connected STA allows direct transitions of several states of the STM, differently to the partially-connected STA which only allows transitions between neighbor states.

## 3 Discussion

Recent clinical studies on VNS for heart failure treatment have failed to show efficacy of the therapy [7–9]. One of the reasons of this failure may be that, in these studies, VNS is delivered in an open loop approach, with manually-configured stimulation parameters. We hypothesize that efficacy of VNS on heart failure, but also in other target applications may be improved by integrating a closed-loop VNS control system, allowing for an adaptive, subject-specific definition of VNS parameters. To our knowledge, the STM based controller presented in this paper is the first closed-loop VNS control systems delivering VNS synchronously to the heartbeat and modifying more than one parameter.

Comparing with control algorithms we have proposed in our previous works (on-off [20], PI [18, 19]), the STM-based controller has, by construction, several advantages: i) no preliminary tuning of control parameters is required, ii) the STM-based controller is suitable for the regulation of a discrete system with non uniform sampling rates and iii) this original approach allowed us to modulate multiple VNS parameters. Moreover, the proposed STM and the corresponding transition algorithm are suitable for the implementation in implantable devices. Indeed, the proposed controller requires limited resources in terms of computing power. Only the rules shown in Fig 4, and one integer addition have to be executed for each loop. Concerning memory usage, each state can be coded with one byte, since less than 256 VNS parameter combinations are used. Finally, the heavier calculations needed during the training phase can be performed using an external computer. These parameters will be fixed on the state table into the device afterwards. This is a straightforward implementation in comparison to a PI controller, which requires a number of floating point operations: additions for the estimation of the integral of the error, two multiplications and at least one last addition to estimate the PI output for each loop. Note also that these operations on the PI only modulate one VNS parameter.

Concerning the characterization of the proposed controller, configurations with different number of states (P_10_ vs P_30_) and transition algorithms (P_30_ vs F_30_) were experimentally evaluated. This experimental evaluation was necessary in this case, since no analytic mathematical characterization can be performed on the proposed controller, due to its non-linear and discontinuous nature and the irregularly sampling rate associated with the cardiac-synchronized function.

Figs 7 and 8 illustrate that it is not possible to obtain a perfectly stable, non-oscillating condition. This is expected in the case of living systems, which are in constant adaptation of their physiological parameters in order to maintain homeostasis through a set of physiological closed-loop systems. The objective is thus to minimize these oscillations and increasing accuracy, while keeping a short settling time. While comparing the P_10_ and P_30_ controllers (Figs 6 and 7 and Table 1), we observed that the MSE is reduced when the number of states is increased (P_30_), at the price of increasing B_*r*_, thus reducing KPI. In fact, according to the STM-based controller definition, all states were sorted in ascending order in the training phase (Fig 5). When the number of states of the partially-connected STM is increased, the resolution in the VNS parameter space of the controller is increased, inducing an improvement of accuracy. However, these partially-connected STM will take at least *N* beats to reach the state *S*_N_. If *N* is increased, the time of convergence is increased too. Increasing the number of states also leads to an improvement of stability (reduced MSE) because RR oscillations decrease with the P_30_ controller.

The influence of the state-transition algorithm was also evaluated by analyzing the performances obtained with partially- and fully-connected STMs using the same number of states (N = 30), on the same sheep for the same targets. By comparing the P_30_ and F_30_ controllers (Figs 6 and 8), we observed a significant reduction on B_*r*_ with the fully-connected STA, while improving the accuracy. In fact, considering a partially- and fully-connected STMs with the same *N* states and *S*_*n*_ the state leading to the minimal error, the partially-connected STM-based controller will take *N* events to reach the state *S*_*n*_ whereas the fully-connected will take around (n/*k*)<n events to reach *S*_*n*_.

Results displayed in Fig 6 summarize the performance associated with each controller. Increasing the number of states in a partially-connected STM-based controller (P_10_ vs P_30_) is associated with an improvement of accuracy, even though the difference is not statistically significant (p > 0.05). The comparison between P_10_ vs. F_30_ shows a significant reduction of MSE (p < 0.05). Moreover, when the number of states in the partially-connected STM-based controller is increased, from *N* = 10 to *N* = 30 states (P_10_ vs. P_30_), B_*r*_ is adversely increased. Nevertheless, the implementation of the fully-connected STA showed the same speed of convergence than the P_10_ controller. The analysis of the performance indicator KPI, on all the controllers, confirms that the proposed F_30_ controller leads to the best compromise between accuracy and speed of convergence.

In addition to the above-mentioned improvements in stability and accuracy, the new fully-connected STM-based controller proposed in this paper also improves speed of convergence with respect to a partially-connected STM. As discussed above, stability and accuracy depend on *N*, while the speed of convergence depends on *k*. These parameters may be chosen during the training phase, according to the required performance and the computation capabilities of the target device. However, a limitation of increasing the number of states is that more memory (in the implantable device) will be required. The complexity of the state transition algorithm remains the same.

Limitations of the study are mainly related to the constraints associated with animal experimentation, since we consider that the analysis of the compromise between the number of states and the accuracy of the controller may be improved by testing the controller for different values of *N*. However, this implies more experimental time, which was limited for this study.

Another limitation of the study concerns the choice of the control variable. In this study we have chosen the RR interval as control variable, since it is the variable used for setting the VNS parameters in the current clinical studies where the VNS is delivered in open loop. However, the proposed controller may be used to regulate other physiological variables representing, for instance, the autonomic nervous system state (heart rate variability markers or sympatho-vagal balance status).

Finally, it should be noted that the proposed STM-based controller integrated a training phase in order to set the VNS parameters corresponding to a given state (*S*_*i*_) and to sort the states in ascending order. Although this training phase is necessary to design the controller specifically to each subject, the state transition model should evolve in order to adapt the therapy both to rapidly changing variables (seconds, minutes, etc..) and slowly changing variables (days, months, years). As a consequence, a strategy should be defined to take into account the temporal multi-resolution application of the proposed closed-loop control approach. In future studies, we plan to further evaluate the robustness of the proposed VNS controllers by activating it for longer periods of time, on non-anesthetized animals, integrating changes in the autonomic state. This will require a fully functional, implantable and chronic version of our system.

## 4 Conclusion

In this work we proposed, implemented and experimentally characterized a new closed-loop VNS control system, based on a state transition model, which allows for the real-time regulation of HR in sheep. By construction, the proposed STM is able to: 1) modulate multiple VNS parameters, 2) regulate a discrete system with non uniform sampling rates, 3) provide an easier subject-specific configuration and 4) be implemented into an implantable device. Results illustrate the interest of using a fully-connected STM and increasing the number of states. The proposed controller (fully-connected STM with *N* = 30) ensures a significant improvement of accuracy and speed of convergence with respect to other STM configurations. In a future work, we will experimentally compare the performance of the proposed controller with respect to a classical PI controller.

## Supporting information

S1 Checklist ARRIVE guidelines checklist. (PDF)

S1 Data These files contains all data underlying the findings of the article. We confirm that there is no identifying information in these files. Datasets containing the WMARR response acquired for each sheep. The data contains: the target, the name of the controller (P10, P30 or F30), RR interval and the current state (CS). All performance indicators are also provided.
(ZIP)
